# PON2 Deficiency Leads to Increased Susceptibility to Diet-Induced Obesity

**DOI:** 10.3390/antiox8010019

**Published:** 2019-01-11

**Authors:** Diana M. Shih, Yonghong Meng, Tamer Sallam, Laurent Vergnes, Michelle L. Shu, Karen Reue, Peter Tontonoz, Alan M. Fogelman, Aldons J. Lusis, Srinivasa T. Reddy

**Affiliations:** 1 Division of Cardiology, Department of Medicine, University of California, Los Angeles, Los Angeles, CA 90095, USA; YMeng@mednet.ucla.edu (Y.M.); TSallam@mednet.ucla.edu (T.S.); AFogelman@mednet.ucla.edu (A.M.F.); Jlusis@mednet.ucla.edu (A.J.L.); SReddy@mednet.ucla.edu (S.T.R.); 2 Department of Human Genetics, University of California, Los Angeles, Los Angeles, CA 90095, USA; lvergnes@ucla.edu (L.V.); KReue@mednet.ucla.edu (K.R.); 3 Department of Integrative Biology and Physiology, University of California, Los Angeles, Los Angeles, CA 90095, USA; michellelshu@ucla.edu; 4 Molecular Biology Institute, University of California, Los Angeles, Los Angeles, CA 90095, USA; 5 Department of Pathology and Laboratory Medicine, University of California, Los Angeles, Los Angeles, CA 90095, USA; PTontonoz@mednet.ucla.edu; 6 Department of Microbiology, Immunology, and Molecular Genetics, University of California, Los Angeles, Los Angeles, CA 90095, USA; 7 Department of Molecular and Medical Pharmacology, University of California, Los Angeles, CA 90095, USA

**Keywords:** obesity, mitochondrial function, antioxidant

## Abstract

(1) Background: Paraoxonase 2 (PON2) is a ubiquitously expressed protein localized to endoplasmic reticulum and mitochondria. Previous studies have shown that PON2 exhibits anti-oxidant and anti-inflammatory functions, and PON2-deficient (PON2-def) mice are more susceptible to atherosclerosis. Furthermore, PON2 deficiency leads to impaired mitochondrial function. (2) Methods: In this study, we examined the susceptibility of PON2-def mice to diet-induced obesity. (3) Results: After feeding of an obesifying diet, the PON2-def mice exhibited significantly increased body weight due to increased fat mass weight as compared to the wild-type (WT) mice. The increased adiposity was due, in part, to increased adipocyte hypertrophy. PON2-def mice had increased fasting insulin levels and impaired glucose tolerance after diet-induced obesity. PON2-def mice had decreased oxygen consumption and energy expenditure. Furthermore, the oxygen consumption rate of subcutaneous fat pads from PON2-def mice was lower compared to WT mice. Gene expression analysis of the subcutaneous fat pads revealed decreased expression levels of markers for beige adipocytes in PON2-def mice. (4) Conclusions: We concluded that altered systemic energy balance, perhaps due to decreased beige adipocytes and mitochondrial dysfunction in white adipose tissue of PON2-def mice, leads to increased obesity in these mice.

## 1. Introduction

Obesity is caused by an excess accumulation of body fat resulting from an imbalance between energy intake and energy expenditure. The incidence of obesity has been rising in the United States and worldwide [1]. Obesity is a risk factor for cardiovascular disease, diabetes, hypertension, and certain types of cancer [1]. In contrast to white adipose tissue (WAT), brown adipose tissue (BAT) dissipates energy by heat generation through UCP1. Upon cold exposure or treatment with β-3 adrenergic agonists, some adipocytes in the WAT called brite or beige adipocytes can acquire brown adipocyte-like features such as increased UCP1 expression [2,3]. Several secretory factors such as catecholamines, FGF21, and irisin have been shown to promote beiging of WAT [3]. Enhanced WAT beiging/browning leads to increased energy expenditure and decreased obesity in both mouse and human studies [4,5]. Interestingly, endoplasmic reticulum (ER) stress-induced mitochondria dysfunction appears to attenuate thermogenic activation/browning of subcutaneous WAT in mice [6], suggesting that ER stress may prevent white to beige conversion and promote obesity.

Furthermore, obesity is associated with adipocyte mitochondrial dysfunction in animal models and humans [7,8,9]. Recent studies have demonstrated a causal relationship between mitochondrial dysfunction and obesity in mice and humans [10,11]. In mice, age-dependent mitochondrial complex IV dysfunction due to loss of essential complex IV components such as Cox5b in white adipocytes led to decreased fatty acid oxidation, increased lipid accumulation, and obesity [10]. In vivo, silencing of Cox5b in the white adipose tissue of young mice led to adipocyte hypertrophy whereas restoration of Cox5b expression led to decreased adipocyte size in aging mice [10]. These findings suggest an important role of mitochondrial complex IV in aging-associated obesity. In humans, specific mutations of the mitofusin 2, a membrane-bound mediator of mitochondrial membrane fusion and inter-organelle communication, caused white adipose tissue mitochondrial dysfunction with increased adipocyte proliferation and survival, leading to obesity [11]. Furthermore, oxidative stress is known to cause mitochondrial dysfunction and metabolic disorders such as insulin resistance and obesity [12,13]. Overexpression of ALCAT1, a lyso-cardiolipin (CL) acyltransferase upregulated by oxidative stress and diet-induced obesity (DIO), increased the synthesis of CL species that are highly sensitive to oxidative damage, leading to mitochondrial dysfunction, reactive oxygen species (ROS) production, and insulin resistance [12]. In contrast, ALCAT1 deficiency prevented the onset of DIO and significantly improved mitochondrial complex I activity, fatty acid oxidation, and insulin signaling in ALCAT1^−/−^ mice [12]. These data demonstrated that increased ROS production can lead to mitochondrial dysfunction and obesity.

Paraoxonase 2 belongs to the paraoxonase gene family. PON2 is ubiquitously expressed and has been localized to nuclear envelope, endoplasmic reticulum, and mitochondria [14,15]. Within the mitochondria, PON2 appears to be localized at the inner mitochondrial membrane where it associates with complex III of the electron transport chain [14]. Overexpression of PON2 in cells prevents ER stress, mitochondrial superoxide formation, cardiolipin peroxidation, and apoptosis [16,17,18], whereas knocking down PON2 level in cells leads to increased ER stress [19]. Previously, we demonstrated that PON2 deficiency in mice leads to increased atherosclerosis due to decreased anti-oxidative and anti-inflammatory capacity [14,20]. PON2 deficiency is associated with impaired mitochondrial aerobic respiration and elevated levels of reactive oxygen species (ROS) in the macrophages [14] and decreased complex I + III activity and increased mitochondrial superoxide level in the liver [14]. In this report, we demonstrated that PON2-def mice are more susceptible to diet-induced obesity. The underlying mechanisms are explored.

## 2. Materials and Methods

### 2.1. Mice and Diet

**Figure 1:**
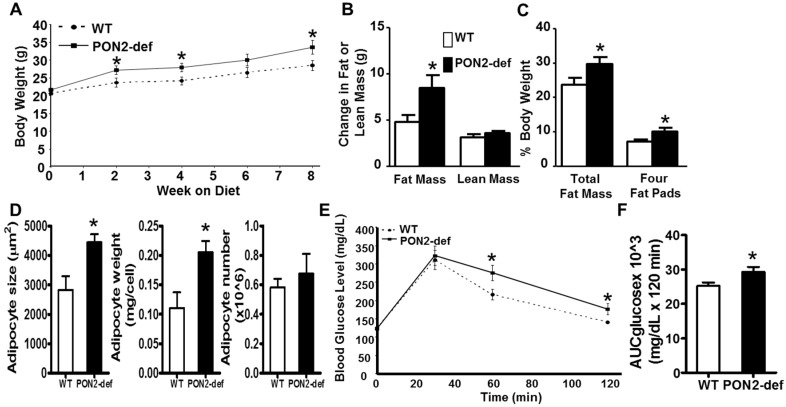
PON2-deficient (PON2-def) mice are more prone to diet-induced obesity and exhibit glucose intolerance. (A) to (D) Two-month-old female PON2-def and wild-type (WT) mice (*n* = 10 for each group) were fed an obesifying diet for 8 weeks before sacrifice. (**A**) Body weights of mice at baseline (week 0), 2, 4, 6, and 8 weeks after the start of obesifying diet feeding are shown. (**B**) Changes in Fat or lean mass after 8 week-feeding of the obesifying diet are shown. (**C**) Total fat mass and weight of four fat pads (gonadal, mesenteric, retroperitoneal, and subcutaneous fat pads), expressed as % of body weight, at sacrifice are shown. (**D**) The mean adipocyte size, weight, and number of subcutaneous fat pads are shown. (**E**) Glucose tolerance test data of WT and PON2-def mice fed the obesifying diet for 7 weeks are shown. (**F**) Mean AUC data of IPGTT are shown. *: *p* < 0.05 vs. WT group.

**Figure 2:**
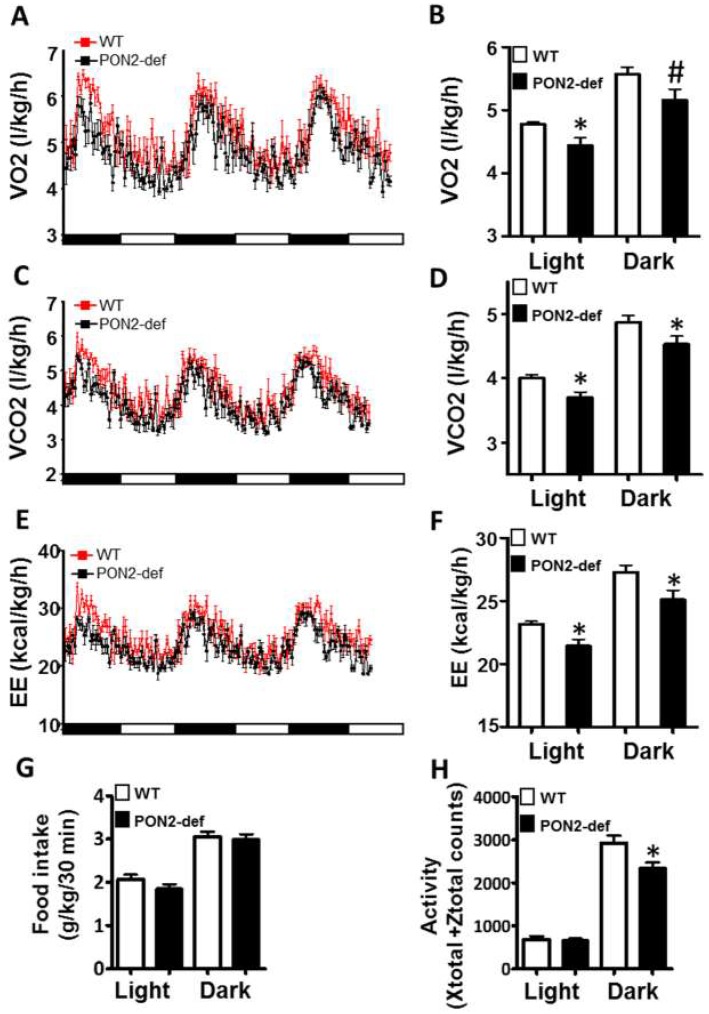
PON2-def mice exhibit altered metabolic phenotypes as compared to the WT mice. Two-month-old female PON2-def and wild-type (WT) mice were fed an obesifying diet for 2 weeks before they were placed individually in metabolic chambers for 3 days to measure metabolic parameters including (**A**,**B**) oxygen consumption rate, (**C**,**D**) CO2 production rate, (**E**,**F**) energy expenditure (EE), (**G**) food intake, and (**H**) activity. *n* = 6 for each group. 12-h light/dark cycles; 72-h total duration; and each light/dark bar represents 12 h duration. After 8 h of acclimation, data from the last 64 h of the 72 h experiment were used for data analysis. #: *p* < 0.07, *: *p* < 0.05, vs. WT group.

**Figure 3:**
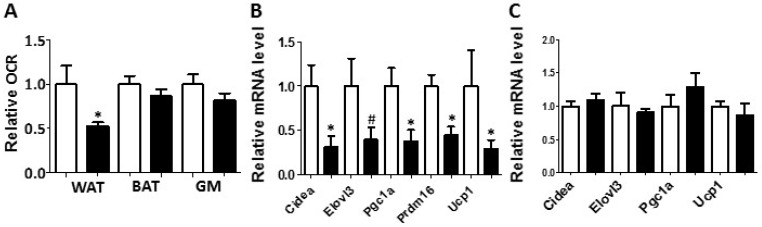
White adipose tissue collected from the PON2-def mice exhibited a decreased oxygen consumption rate and decreased mRNA levels of gene markers for beige adipocytes. (**A**) Relative oxygen consumption rates of subcutaneous fat pads (WAT), brown adipose tissue (BAT) and gastrocnemius muscle (GM) isolated from mice fed the obesifying diet for 8 weeks are shown. *n* = 6 for each group. Gene expression analyses of subcutaneous fat pads (**B**), and BAT (**C**) collected from mice fed the obesifying diet are shown. *n* = 6 for each group. #: *p* = 0.085, *: *p* < 0.05, vs. WT group.

All animal experiments were approved by the UCLA Animal Care and Use Committee, in accordance with PHS guidelines. The protocol number is ARC # 1992–169 (Approval Period from 6/6/2016 through 11/25/2018). The mice used for the study were derived originally from the offspring of PON2 heterozygous mice intercross. PON2-def mice that resulted from the intercross were used to establish the PON2-def colony, whereas wild-type (WT) mice that resulted from the intercross were used to establish the WT colony. Two-month-old female PON2-def and wild-type (WT) mice were fed an obesifying diet (D12266B, Research Diets) for 8 weeks and fasted overnight before blood and tissue collection. Two cohorts of mice were investigated in this study. The overview of experimental procedures is shown in Supplemental Figure S1. For the obesity experiment presented in Figure 1 and Table 1, 10 mice per genotype group were used (Cohort #1). This was based on power/sample size calculation assuming a 40% difference in body weight between the two groups of mice, common standard deviation: 30%, type I error rate: 0.05, and power: 0.8. For data presented in Figure 2 and Figure 3, a separate cohort (cohort #2, Supplemental Figure S1) was used.

### 2.2. Plasma Lipids, Glucose, and Insulin Assays

Plasma triglyceride, total cholesterol, high density lipoprotein (HDL) cholesterol, free fatty acid, and glucose levels were measured by colorimetric assays as previously described [21]. Plasma insulin levels were measured by ELISA using kits purchased from ALPCO (Salem, NH, USA).

### 2.3. Body Composition by Quantitative Nuclear Magnetic Resonance

Animals were measured for total body fat mass and lean mass by nuclear magnetic resonance (NMR) using the Bruker Minispec with software from Echo Medical Systems (Houston, TX, USA) [22]. 

### 2.4. Glucose Tolerance Test

Mice were fed the obesifying diet for 7 weeks before intraperitoneal glucose tolerance tests (IP-GTT, 1g glucose/kg body weight) were performed after 16-hour fasting [23]. Area under the curve was calculated using Prism 5 (GraphPad Software Inc., La Jolla, CA, USA)

### 2.5. Metabolic Chamber Study

Mice were fed the obesifying diet for 2 weeks before metabolic chamber study was performed using a Columbus Instruments Comprehensive Lab Animal Monitoring System (Columbus Instruments International, Columbus, OH, USA) as previously described [24].

### 2.6. Oxygen Consumption Study

The oxygen consumption rate of tissue was determined using an XF24–3 Extracellular Flux Analyzer (Seahorse Bioscience, Billerica, MA, USA) as described [21].

### 2.7. RNA Isolation and Quantitative RT-PCR Analyses

Total RNA samples from tissues were isolated using Trizol reagent (Life Technologies, Carlsbad, CA, USA) according to the manufacturer’s protocol. The cDNA was synthesized using the High Capacity cDNA Reverse Transcription Kit (Applied Biosystems, Foster City, CA, USA). Quantitative PCR was performed using gene-specific primers (Supplemental Table S1) and the Roche SYBR green master mix in a Roche Lightcycler 480 system (Roche, Indianapolis, IN, USA). The mRNA levels of specific genes were normalized to the mRNA levels of the housekeeping gene, Rpl13a, of the same sample.

### 2.8. Determination of Average Adipocyte Size and Adipose Cell Number

H & E stained histological sections of subcutaneous fat pads were used for determination of adipocyte size as previously described [25]. Determination of adipocyte number was performed as described [26].

### 2.9. Statistical Analysis

Two tailed, unpaired Student’s t test, available in Prism 5 (GraphPad Software Inc., La Jolla, CA, USA), was used for statistical analysis to compare group means between the WT and PON2-def mice.

## 3. Results and Discussion

### 3.1. PON2-def Mice Are More Susceptible to Diet-Induced Obesity and Exhibit Glucose Intolerance

Female PON2-def mice exhibited similar body weight (Figure 1A) and adiposity (as judged by % fat mass/body weight, data not shown) as compared to the age and sex matched wild-type mice. However, when placed on a high fat, high sucrose obesifying diet, the PON2-def mice weighed significantly more at 2, 4, and 8 weeks after feeding of the obesifying diet (Figure 1A). PON2-def mice gained significantly more fat mass but the same amount of lean mass as compared to the WT mice after 8-week feeding (Figure 1B). PON2-def mice exhibited significantly increased adiposity as expressed by both % fat mass/body weight and % weight of four major fat pads/body weight (Figure 1C). The gonadal and subcutaneous fat pads of the PON2-def mice were significantly larger as compared to those of the WT mice (*p* < 0.05 for both fat pads, data not shown), whereas the mesenteric and retroperitoneal fat pads of the PON2-def mice showed a trend of increased weights (*p* = 0.06 and *p* = 0.08, respectively, data not shown) as compared to those of the WT mice. The mean adipocyte size and weight, but not adipocyte number, of subcutaneous fat pads of the PON2-def mice were significantly increased as compared to those of the WT mice (Figure 1D), suggesting that adipocyte hypertrophy can explain, in part, the increased obesity observed in the PON2-def mice. There are no significant differences in plasma levels of triglyceride, total cholesterol, HDL cholesterol, free fatty acid, and glucose between the WT and PON2-def mice (Table 1). However, the fasting insulin levels of the PON2-def mice after 8 weeks of diet feeding were significantly increased as compared to those of the WT mice (Table 1, *p* < 0.05). Glucose tolerance test revealed that PON2-def mice had significantly increased blood glucose levels at 60 min, and 120 min after receiving 1g/kg dose of glucose as compared to the WT mice (Figure 1E). The mean area under the curve (AUC) from the IPGTT of the PON2-def mice was significantly increased as compared to the WT mice (Figure 1F), suggesting glucose intolerance in the PON2-def mice. 

### 3.2. PON2-def Mice Exhibit Altered Systemic Energy Balance and a Decreased Oxygen Consumption Rate in the White Adipose Tissue

Metabolic chamber study showed that PON2-def mice exhibited a significantly decreased oxygen consumption rate (OCR) in the light period and a trend of decreased OCR in the dark period as compared to the WT mice (Figure 2A,B). The CO_2_ production rates of the PON2-def in both the light and dark periods were significantly decreased (Figure 2C,D) as compared to the WT mice. Interestingly, PON2-def mice showed significantly decreased energy expenditure during both light and dark periods as compared to the WT mice (Figure 2E,F). The food intake was similar between the two groups of mice (Figure 2G). Furthermore, PON2-def mice were less active during the dark periods (Figure 2H). We then investigated the oxygen consumption rates of tissues collected from the PON2-def and WT mice. The oxygen consumption rate of subcutaneous fat pads isolated from the PON2-def mice was more than 50% lower as compared to those of the WT mice (Figure 3A). On the other hand, no significant differences were observed in oxygen consumption rates between the brown adipose tissues and gastrocnemius muscles of PON2-def and WT mice (Figure 3A).

### 3.3. Gene Expression Analysis of WAT and BAT

Enhanced WAT beiging leads to increased energy expenditure and decreased obesity in both mouse and human studies [4,5]. We hypothesized that the increased obesity observed in the PON2-def mice might be partly due to decreased beiging of WAT. Gene expression analysis of the subcutaneous WAT showed that the expression levels of beige adipocyte gene markers including Cidea, Pgc1a, Prdm16, and Ucp1 were significantly decreased by more than 50% in the PON2-def as compared to those of the WT mice (Figure 3B), providing evidence that PON2 deficiency is associated with deceased beiging of WAT. In contrast, the expression levels of characteristic brown fat genes including Cidea, Elovl3, Pgc1a, and Ucp1 in the BAT of PON2-def and WT mice were similar (Figure 3C). 

## 4. Conclusions

Our study demonstrated that PON2 deficiency leads to increased DIO and impaired glucose tolerance in mice. The PON2-def mice exhibited decreased energy expenditure and oxygen consumption with no change in food consumption as compared to the WT mice, suggesting that decreased energy expenditure may be the underlying cause of obesity observed in the PON2-def mice. The oxygen consumption rate of the subcutaneous WAT isolated from the PON2-def mice was significantly decreased as compared to the WT mice. Furthermore, the expression of beige cell gene markers was significantly lower in the subcutaneous WAT of PON2-def mice. These findings suggest that decreased beige adipocyte number and impaired mitochondrial function may contribute to the increased DIO associated with PON2 deficiency. Previous studies have demonstrated that PON2 plays an important role in preventing ER stress, mitochondrial superoxide formation, cardiolipin peroxidation, and apoptosis in cultured cells [16,17,18,19]. In agreement, our groups have shown that PON2 deficiency was associated with impaired mitochondrial aerobic respiration and elevated levels of reactive oxygen species (ROS) in macrophages [14] and decreased complex I + III activity and increased mitochondrial superoxide level in the livers [14] of PON2-def mice. Therefore, we conclude that the increased ER stress and mitochondrial dysfunction associated with PON2 deficiency are likely the underlying mechanisms leading to decreased beiging and energy expenditure, and increased adipocyte hypertrophy and obesity observed in the PON2-def mice. 

## Figures and Tables

**Table 1:** Plasma lipid, glucose, and insulin levels of WT and PON2-def mice fed the obesifying diet for 8 weeks.

Genotype	*n*	Triglyceride	Total Chol.	HDL Chol.	VLDL/IDL/LDL Chol.	Unesterified Chol.	Free Fatty Acid	Glucose	Insulin
WT	9	35 (7)	128 (5)	91 (4)	47 (12)	18 (1)	48 (3)	143 (10)	336 (48)
PON2KO	10	34 (8)	118 (17)	87 (13)	30 (6)	19 (2)	47 (3)	147 (8)	485 (47)
*p* value		0.91	0.58	0.80	0.20	0.63	0.76	0.71	0.04

Values shown are means and (standard errors). Units are mg/dL except insulin which is shown in pg/mL. Abbreviations: Chol.: cholesterol, VLDL/IDL/LDL: very low density lipoprotein/intermediate density lipoprotein/low density lipoprotein.

## Supplementary Materials

The following are available online at http://www.mdpi.com/2076-3921/8/1/19/s1, Figure S1. Overview of the experimental procedures. Table S1. qPCR primers used in the study.
