# Supplementary material for: PON2 Deficiency Leads to Increased Susceptibility to Diet-Induced Obesity
Source: Antioxidants (Basel). 2019 Jan 11;8(1):19. doi: 10.3390/antiox8010019 (PMC6356528; doi:10.3390/antiox8010019)
Supplement: Supplementary file 1 [file antioxidants-08-00019-s001.pdf]

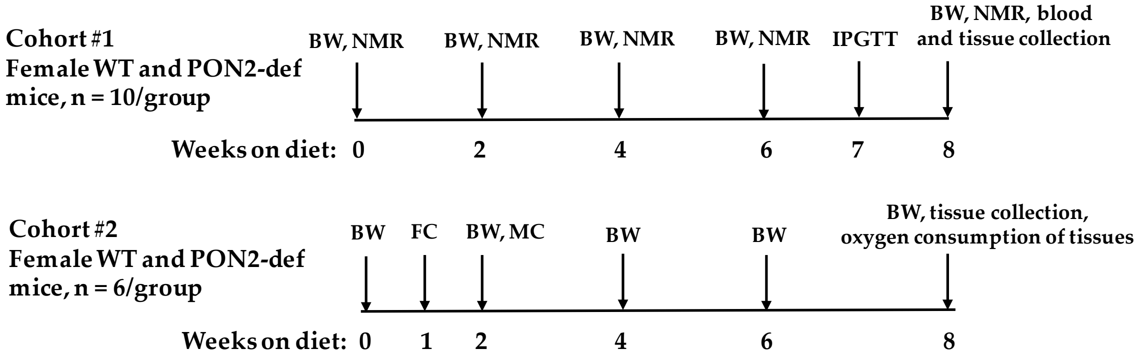

Figure 1. Overview of the experimental procedures. Two cohorts of female WT and PON2-def mice were investigated in this study. For cohort #1, 2-month-old female WT and PON2-def mice (n = 10 per group) were fed an obesifying diet (D12266B, Research Diets) for 8 weeks before euthanasia. The phenotypes collected include body weight (BW) and NMR for body composition at 0, 2, 4, 6, and 8 weeks; intraperitoneal glucose tolerance test (IPGTT) at week 7; weights of four major fat pads, gonadal, mesenteric, retroperitoneal, and subcutaneous fat pads, adipocyte size, weight, and number of subcutaneous fat pads, plasma lipid, glucose, and insulin levels at week 8. Data presented in Figure 1 and Table 1 are from cohort #1. For cohort #2, 2-month-old female WT and PON2-def mice (n = 6 per group) were fed an obesifying diet (D12266B, Research Diets) for 8 weeks before euthanasia. The phenotypes collected include body weight (BW) at 0, 2, 4, 6, and 8 weeks; daily food consumption between week 1 and week 2; metabolic chamber (MC) study at week 2, oxygen consumption rate of WAT, BAT, and skeletal muscle at week 8. Gene expression analysis of subcutaneous fat pads and BAT at week 8. Data presented in Figures 2 and 3 are from cohort #2.

Table S1. qPCR primers used in the study.

Name	Sequence
**Cidea-F**	AACCATGACCGAAGTAGCCG
**Cidea-R**	CCAGGCCAGTTGTGATGACT
**Elovl3-F**	TCCGCGTTCTCATGTAGGTCT
**Elovl3-R**	GGACCTGATGCAACCCTATGA
**Pgc1a-F**	TGAAAAAGCTTGACTGGCGTC
**Pgc1a-R**	CCGATTGGTCGCTACACCAC
**Prdm16-F**	GCCATGTGTCAGATCAACGA
**Prdm16-R**	CCTTCTTTCACATGCACCAA
**Rpl13a-F**	CCCTCCACCCTATGACAAGA
**Rpl13a-R**	TTCTCCTCCAGAGTGGCTGT
**Ucp1-F**	GGCCTCTACGACTCAGTCCA
**Ucp1-R**	TAAGCCGGCTGAGATCTTGT
